# Gluten-containing flours and gluten-free flours as a source of calcium, magnesium, iron and zinc

**DOI:** 10.1038/s41598-024-65530-2

**Published:** 2024-06-25

**Authors:** Iwona Mystkowska, Ewa Plażuk, Adam Szepeluk, Aleksandra Dmitrowicz

**Affiliations:** 1 Department of Dieteties, John Paul II University in Biala Podlaska, Sidorska Street 95/97, 21-500 Biala Podlaska, Poland; 2 Regional Research Centre On Environment, Agriculture and Innovative Technologies, EKO-AGRO-TECH, John Paul II University in Biala Podlaska, Sidorska 95/97, 21-500 Biala Podlaska, Poland; 3 Department of Nursing, John Paul II University in Biala Podlaska, Sidorska Street 95/97, 21-500 Biala Podlaska, Poland

**Keywords:** Calcium, Iron, Magnesium, Gastrointestinal diseases, Coeliac disease, Malnutrition

## Abstract

Wheat flour is widely used in Poland for the preparation of bread, pasta and other foods. Due to the increasing number of people diagnosed with diet-related diseases, consumer awareness of health-promoting issues and interest in gluten-free products (GFP). There is a dynamic development of the market for these foods with high quality and nutritional value and minerals that benefit human health and prevent deficiencies in patients on a gluten-free diet. The aim of this study was to determine the content of minerals: Ca, Fe, Mg and Zn in flours using the ICP-OES method. The mineral composition of selected GF flours available on the Polish market was analysed. It was tested how they supplement the mineral requirements compared to gluten-containing flours. It was found that these products can be a valuable source of essential minerals, which are often in short supply, especially in patients with gastrointestinal disorders. As our study has shown, flours from the GFP group are a good source of essential minerals, especially in the case of chia and flax flours, as well as buckwheat, amaranth, quinoa, lupin or almonds flours.

## Introduction

Grain products are a staple of our diet, as we eat them every day in the form of various types of bread, pasta, groats and pizza. The main ingredient in these foods is wheat flour—long the main source of carbohydrates in the Western world and more recently in Eastern societies^1,2^. This can be a cause for concern, particularly because of gluten, which has been linked to the development of some modern chronic diseases. Gluten is found in many plant products and foods prepared with them, which are derived from grains such as: wheat, rye and oats. This causes problems for people with gluten intolerance, who especially need to pay attention to what they consume. As gluten makes up 80% of modern wheat proteins and is also used extensively as a food additive in the food industry, there is no doubt that its consumption is growing rapidly. In parallel, there is an increased incidence of many autoimmune diseases (AD) that have become popular in recent decades. Consumption of such gluten-containing foods can cause damage to the small intestinal mucosa in patients, which contributes to impaired absorption of nutrients and minerals and also leads to many systemic complications, such as anemia, rickets, osteoporosis, growth disorders and reduced weight gain^3–6^.

The gluten-free diet (GFD) is the most popular elimination diet, which can also often be due to the trend to eat healthier whole-food equivalents of "gluten-free" foods, not always due to the presence of gluten-related disorders^7,8^. Although GFD is associated with healthy eating, studies indicate nutritional imbalances in long-term users^9^. These include nutrients, vitamins and fiber, as well as macronutrients and micronutrients, primarily minerals: Ca, Fe, Mg and Zn^10,11^. Data on minerals in gluten-free products are still limited, research on improving the nutritional quality of GFP is warranted, as mentioned by many researchers^11–13^.

Products obtained from seeds can be divided into two main categories—milled products and products processed into flour. Milled products include mechanically milled cereal raw materials that contain grain fractions in a less fine form than flour. Products processed into flour, on the other hand, are cereal grains that have been ground into a fine powder and—depending on whether whole grain was used for grinding—may be whole grain or—in the case of refined grain—refined. During flour production, especially refining, the outer layer of the grain is removed, leaving only the inner part^10,14^. Our study focused on the analysis of different flours, both gluten-containing and gluten-free. All products used in the study were purchased as already processed into flour, none of which we milled.

The purpose of the study was to determine the mineral content: Ca, Fe, Mg and Zn in gluten-free flours gaining popularity in Poland as an alternative to gluten products, comparatively compared to popular flours containing gluten. We focused mainly on assessing the content of selected elements that are relevant to assessing product quality and meeting basic food standards. Currently, a full range of gluten-free grain food preparations is available in our country, for the manufacture of which naturally gluten-free raw materials are used, such as rice, quinoa, corn, buckwheat, millet, amaranth, tapioca, lupin, almonds, chestnuts, flax or chia and others. They can be used as additives to so-called daily bread or other dishes in the form of flours rich in nutritional value, fiber and elements: macro and microelements, so it was important to check their content for these key minerals. Our aim was to provide a comprehensive assessment of the mineral composition of the product, which may be relevant for the average consumer.

## Materials and methods

The study material consisted of finished products processed into flour purchased in local grocery stores in Poland in the years 2022–2023 before their expiration date. Flours from gluten-containig (n = 43) and gluten-free seeds (n = 13) were purchased. Among the gluten-containing flours, the following groups were distinguished: refined rye flours (n = 3), rye wholemeal flours (n = 3), refined wheat flours (n = 29), wholemeal wheat flours (n = 2), spelt wholemeal flours (n = 3), spelt refined flour (n = 1), einkorn flour (n = 1) and emmer flour (n = 1). On the other hand, of the gluten-free flours were tested: millet flour (n = 3), buckwheat flour (n = 1), maizeflour (n = 1), sorghum flour (n = 2), chestnut flour (n = 1), amaranth flour (n = 1), almond flour (n = 1), lupin flour (n = 1), tapioca flour (n = 1) chia flour (n = 1), quinoa flour (n = 2) and flax flour (n = 2). All flours were stored in a dry, dark and cool place prior to testing, and the information on the packaging confirmed that the flours met all food approval standards. The accepted number of samples in our work was determined by the realistic purchasing capacity of the average consumer. The consumers perspective and their accessibility to the product were crucial for the representativeness of our study. The total ash content was determined as a non-combustible residue obtained after incineration in an oven (Magma-Therm, DANLAB, Poland) of the sample according to AACC Method 08–01^15^ and the moisture content of the flours using a dryer (Type SLN 240, POL-EKO Poland) at 130 °C according to PN-EN ISO 712^16^. Before analysing the mineral content, the flours were first dried at 65 °C, and about 0.5 g weighed. All samples were mineralized with 65% nitric acid V and 36–38% hydrochloric acid (6:1). Mineralization was carried out in a microwave digestion system (AantonPaar, Austria) until the sample was completely digested. The solution was then filtered and diluted with distilled water to a solution volume of 50 ml. The content of selected macro (Ca and Mg) and micronutrients (Fe and Zn) was determined by inductively coupled plasma optical spectrophotometry using an ICP-OES (Inductively Coupled Plasma—Optical Emission Spectrometry) spectrophotometer (Spectroblue, AMETEK Inc., Germany). Before analysing the samples, certified standard VHGSM68-1-500 Element Multi Standard 1 with 48 control elements in 5% HNO_3_ solution was used. The collected material was subjected to statistical analysis using STATISTICA version 13.0 software from StatSoft Poland. should be noted that the study may be prone to systematic errors that could have caused theresults to differ from the actual results. There is a possibility that environmental or technical factors may have influenced the results of the study, which was not in the analysis. We attempted to control for all confounding factors to minimize the risk of error, but we cannot rule out their existence. The data were expressed as the median content of selected elements in different flours. They were then subjected to hierarchical cluster analysis (HCA) using Ward's method agglomeration and Euclidean distance. The Kruskal–Wallis test was used for statistical analysis of the data. Statistical inference was performed with a standardized significance level of α = 0.05.

## Results and discussion

**Figure 1:**
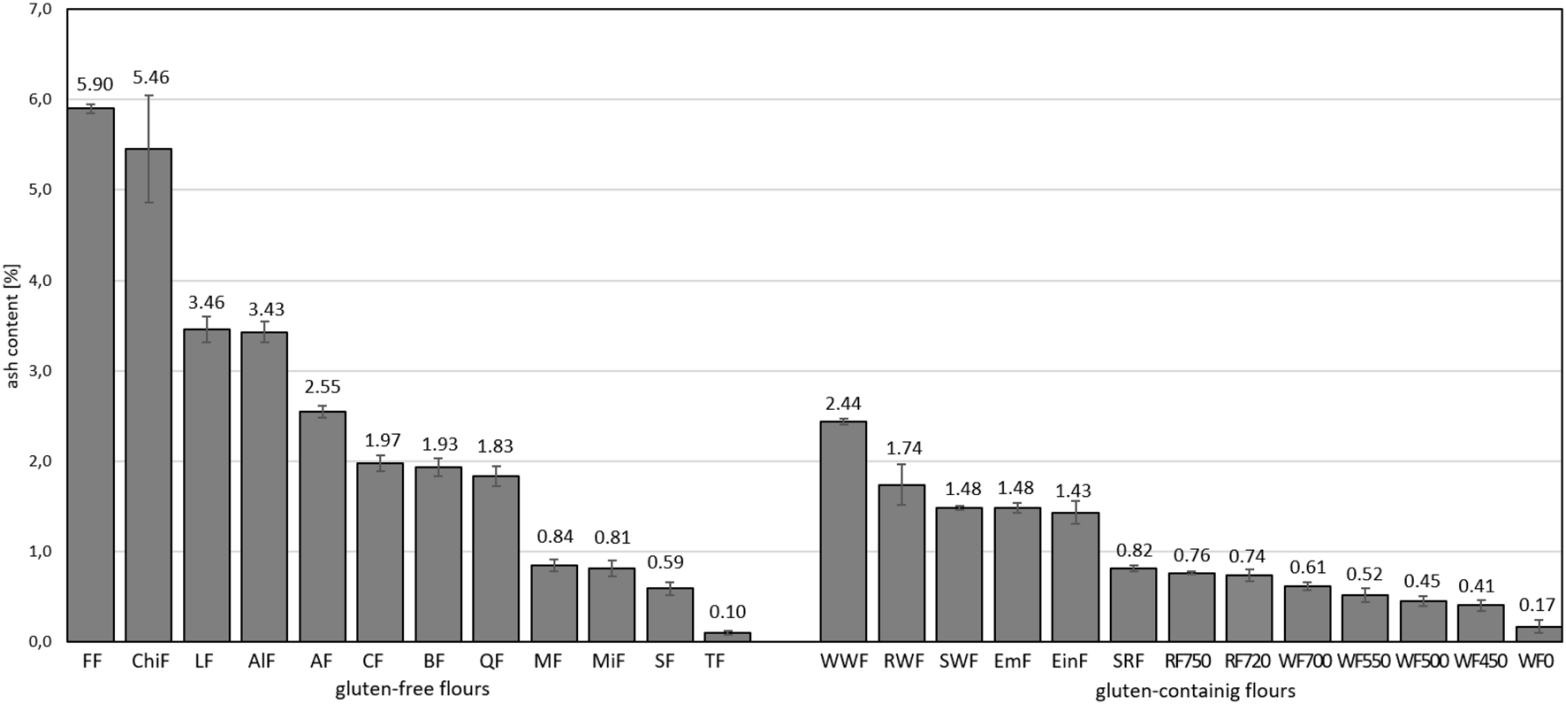
Ash content of gluten-free and gluten-containing flours. Designations for gluten-free flours: flax flour (FF), chia flour (ChiF), lupin flour (LF), almond flour (AlF), amaranth flour (AF), chestnut flour (CF), maize flour (MF), millet flour (MiF), buckwheat flour (BF), quinoa flour (QF) sorghum flour (SF), tapioca flour (TF). Designations for gluten flours: wholemeal wheat flours (WWF) and wholemeal rye flours (RWF) Type 2000, refined rye flours (RF) Type 720 and 750, refined wheat flours (WF) Type: 00, 450, 500, 550 and 700, wholemeal spelt flours (SWF), refined spelt flours (SRF), einkorn flours (EinF), emmer flours (EmF).

Wholemeal flour consists of endosperm, germ and bran, whereas refined flour contains only the endosperm, as the germ and bran are removed during the milling process. Due to the presence of bran and germ, wholemeal flour has a higher ash content. It is also characterized by a higher content of fiber, vitamins and minerals and usually has a lower glycaemic index^10,14^.In the analysis of the ash content of the flours tested, a varying degree of total ash was found, which made it possible to classify the samples tested into flours that were more and less purified in the processing to flour. The ranges for gluten-free flours and gluten-containing flours—whole grains and refined cereals, i.e. without the outer parts of the grain, are shown in Fig. 1.

Based on the ash content ranges given for different flours (Fig. 1), some conclusions can be drawn about their classification as whole grain or refined. However, it should be borne in mind that this classification can also depend on other factors, such as the production process or possible purification. Whole grain products include flaxseed flour, which has a very high ash content, indicating that it contains whole flaxseeds. In addition, chia flour, lupin flour, almond flour, amaranth flour, chestnut flour, buckwheat flour and quinoa flour also have a high ash content, suggesting that they contain more of the plant's nutrients, which is a characteristic of whole grain products. Wholemeal flours containing gluten from: Wheat, rye, spelt, einkorn and emmer flours show the highest values in the determination of total ash. Wheat flours of type 00—550 have the lowest ash content, which indicates that the grains used for their production are the most refined. In contrast, gluten-free flours made from maize, millet and sorghum have a similar ash content to cereal flours of type 500—750. Tapioca flour, like type 00 wheat flour, can also be refined during processing as it has a low ash content (Fig. 1).

Minerals can be divided into major and those found in trace amounts in the human body. Macronutrients include elements present in the human body in amounts greater than 0.01% of body weight. Among them are calcium, potassium, phosphorus, magnesium, sodium and chlorine. Micronutrients account for less than 0.01% of body weight and include, among others: iron, manganese, copper, zinc, iodine and selenium. Below is information on the content in ready-made foodstuffs in the form of flour of selected macronutrients (Ca and Mg) and micronutrients (Fe and Zn).

Table 1 shows the mineral content of the gluten and gluten-free flours analyzed. The content of Ca and Mg, as well as Fe and Zn, varied significantly depending on the type of product. It is worth noting that the nutrient content of flours due to plant origin depends on genetic factors, such as plant variety, as well as environmental factors—the type of soil, its moisture content and climatic conditions. The products representing our samples are flours of various origins, which have been approved for marketing in Poland. The water content in the flours tested ranged from 11.6 to 13.9%, which is below the permissible value (14.5%) according to the standard . All flours were stored in a dry, dark and cool place before being tested. In addition, the information on the packaging confirmed that the flours met the food approval standards.

**Table 1:** Median minerals in individual flours ± standard deviation (mg/100 g of product).

		Ca	Mg	Fe	Zn
Gluten-containig flours	Rye refined flour	45.4 ± 12.4	38 ± 7.51	4.01 ± 1.52	1.47 ± 0.41
	Rye wholemeal flour	70.2 ± 19.5	30.2 ± 4.27	5.03 ± 1.15	3.25 ± 0.54
	Wheat refined flour	34.0 ± 0.91	25.2 ± 8.79	2.10 ± 0.95	0.76 ± 0.03
	Wheat wholemeal flour	45.2 ± 19.0	105.5 ± 31.4	4.77 ± 0.35	2.73 ± 0.10
	Spelt refined flour	29.0 ± 0.11	35.0 ± 0.30	1.87 ± 0.05	1.33 ± 0.05
	Spelt wholemeal flour	25.2 ± 0.44	70.1 ± 36.8	2.32 ± 1.24	2.07 ± 1.12
	Einkorn wholemeal flour	61.4 ± 0.19	100.3 ± 0.15	4.01 ± 0.01	3.51 ± 0.01
	Emmer wholemeal flour	28.2 ± 0.11	86.7 ± 0.47	2.71 ± 0.09	2.38 ± 0.01
Gluten-free flours	Millet flour	13.2 ± 0.57	85.5 ± 13.5	2.64 ± 0.45	2.47 ± 0.57
	Buckwheat flour	17.0 ± 0.13	233.6 ± 0.11	2.54 ± 0.01	2.22 ± 0.01
	Maize flour	13.9 ± 0.05	79.8 ± 0.15	2.02 ± 0.01	1.73 ± 0.26
	Sorghum flour	10.28 ± 0.19	54.1 ± 4.10	1.65 ± 0.49	0.99 ± 0.01
	Chestnut flour	39.8 ± 0.13	64.6 ± 0.27	1.18 ± 0.05	1.03 ± 0.03
	Amaranth flour	74.9 ± 0.12	211.1 ± 0.52	4.24 ± 0.01	2.04 ± 0.03
	Almond flour	170.4 ± 0.23	229.8 ± 0.14	2.21 ± 0.01	3.23 ± 0.01
	Lupin flour	142.8 ± 0.78	166.3 ± 0.65	3.33 ± 0.01	2.46 ± 0.05
	Tapioca flour	26.9 ± 0.03	14.60 ± 0.10	0.59 ± 0.01	0.12 ± 0.001
	Quinoa flour	36.45 ± 1.62	147.8 ± 36.5	4.58 ± 2.71	2.38 ± 0.47
	Flax flour	252.9 ± 10.2	590.8 ± 73.3	13.29 ± 9.77	6.44 ± 0.01
	Chia flour	530.4 ± 0.37	499.2 ± 0.14	6.63 ± 0.05	6.11 ± 0.05

Based on the data presented (Table 1), it can be concluded that the calcium content of the flours studied varies within a wide range from 10.28 to 530.4 mg/100 g. Chia flour is distinguished by its unusually high content of the macronutrient compared to flours derived from other plants albo seeds grainsand pseudo-grains. According to our results, in the mineral composition of this food product, Ca was the macronutrient present at 530.4 mg/100 g, which corresponds with the results of Dutra et al.^17^ and Barreto et al.^18^, who reported a similar value for calcium (525 and 566.6 mg/100 g), while the United States Department of Agriculture (USDA) reported a higher value (631 mg/100 g) for this element^19^. In contrast, Goyat et al. reported that the product contains as much as 692.91 ± 1.39 mg/100 g^20^. This is also confirmed by other studies, where the range of Ca concentrations is wide, from 460 to 671 mg/100 g^21,22^. In second place in terms of calcium content is flax (Table 1) 252.85 mg/100 g, which is in line with the values determined by the USDA and others (220–380 mg/100 g)^19,21,23,24^. Differences in plant mineral compositions can be seen depending on variety, regional characteristics (soil type, rainfall) and/or fertilizer use^25^. Significant amounts of calcium are contained in almond flour—more than 170 mg/100 g of the product (Table 1), although the USDA National Nutrient Database for Standard Reference indicates that as much as 232 mg of this element is contained in 100 g of the product^19^. Distinguished among gluten-free flours, amaranth flour also has a high content of this element (74.90 mg/100 g) (Table 1). More than twice as high values of this element were recorded by other researchers in amaranth, ranging from 159 to 186 mg/100 g^19,26–28^. In contrast, the lowest calcium contents can be indicated in GF flours: millet, sorghum and corn flours (Table 1), as also indicated by Kunachowicz^26^.

The Ca content of buckwheat flour varies widely depending on the source cited. Ikeda et al. estimated the content of the macronutrient as 20.3 ± 5.4 mg/100 g^29,30^, a result similar to that obtained in our study (Table 1), USDA database reports a value of 21 mg/100 g, while Kunachowicz reports a 2 times higher Ca concentration in buckwheat flour—40 mg/100 g^26^. The results for chestnut flour (39.8 ± 0.13 mg/100 g) are lower than the value suggested by the USDA (56 mg/100 g^19^, while those for sorghum flour (10.28 ± 0.19 mg/100 g) are close to the value suggested by the literature (11 mg/100 g)^19^. Similarly for quinoa flour, for which the results of the element content (36.45 ± 1.6 mg/100 g) are only slightly lower than those reported by the USDA (38 mg/100 g)^19^. In contrast, the lowest calcium contents can be indicated in GF flours: millet and corn flours (Table 1), as also indicated by Kunachowicz 13 and 7.4 mg/100 g of these products^26^.

As for gluten-containig flours, the amount of calcium is significant in whole grain flours-especially rye and einkorn flour, and half as much in wheat flour, which is several times lower than in some of the GF flours with the highest calcium concentrations (Table 1), which correlates with the results of Fernández-Canto et. al^31^, while others report lower Ca concentrations in these flours^26,32^. Kunachowicz found that Ca content in rye flour occurs at 19–37 mg/100 g of product, depending on the type of flour^26^. Similar contents were also shown by Ertl and Goessler, with rye flour containing 0.29 ± 0.03 g Ca /kg, and wholemeal rye flour containing 0.37 ± 0.04 g/kg, corresponding to 29 and 37 mg/100 g of product, respectively^32^. Based on the analysis (Table 1), it was found that the content of this macronutrient in flours derived from wheat and spelt wheat occurs at the level of 25.2–45.2 mg/100 g, depending on the type of grain and production method. These values are slightly higher than those shown by Ertl and Goessler's team of 0.19 ± 0.03 g/kg in wheat flour and 0.39 ± 0.08 g/kg in wholemeal wheat flour, and 0.26 ± 0.01 g/kg and 0.40 ± 0.01 g/kg in spelt flour—refined and wholemeal, respectively^32^. On the other hand, Fernández-Canto et al. determined the calcium concentration in the wheat wholemeal flour mixture as 73.0 ± 2.7 mg/100 g^31^. The results for refined spelt flour (29.0 ± 0.11 mg/100 g) and wholemeal spelt flour (25.2 ± 0.44 mg/100 g) are also comparable to literature data (30–40 mg/100 g^19,32^ for wholemeal and 26 mg/100 g for wholemeal^32^. According to Table 1, of all wheat flours, it is einkorn that has the highest Ca content. Hidalgo and Brandolini^33^ also highlight the much higher levels of this element in einkorn wheat than traditional wheat, just as Rachoń et al. found the highest levels of macronutrients just in einkorn, compared to other wheat^34^. It should be taken into account that the most common macronutrient deficiencies reported in the literature are calcium and magnesium deficiencies^35,36^. With regard to calcium, controversial results can be found depending on the reference intake values in different countries. Reduced levels of: iron, folic acid, vitamin B12, vitamin D and also magnesium have been documented in most patients with celiac disease (CD)^37^. Recommended limits for macronutrients and micronutrients in food have been set by various health and food safety agencies around the world^21^. In accordance with the Regulation of the European Parliament and of the Council of October 25, 2011, daily reference intake values (abbreviated as RDA) for vitamins and minerals have been defined^38^. They are only indicative values and are intended as a reference for a healthy adult. These values do not take into account varying factors such as physical activity or human height and weight.

**Figure 2:**
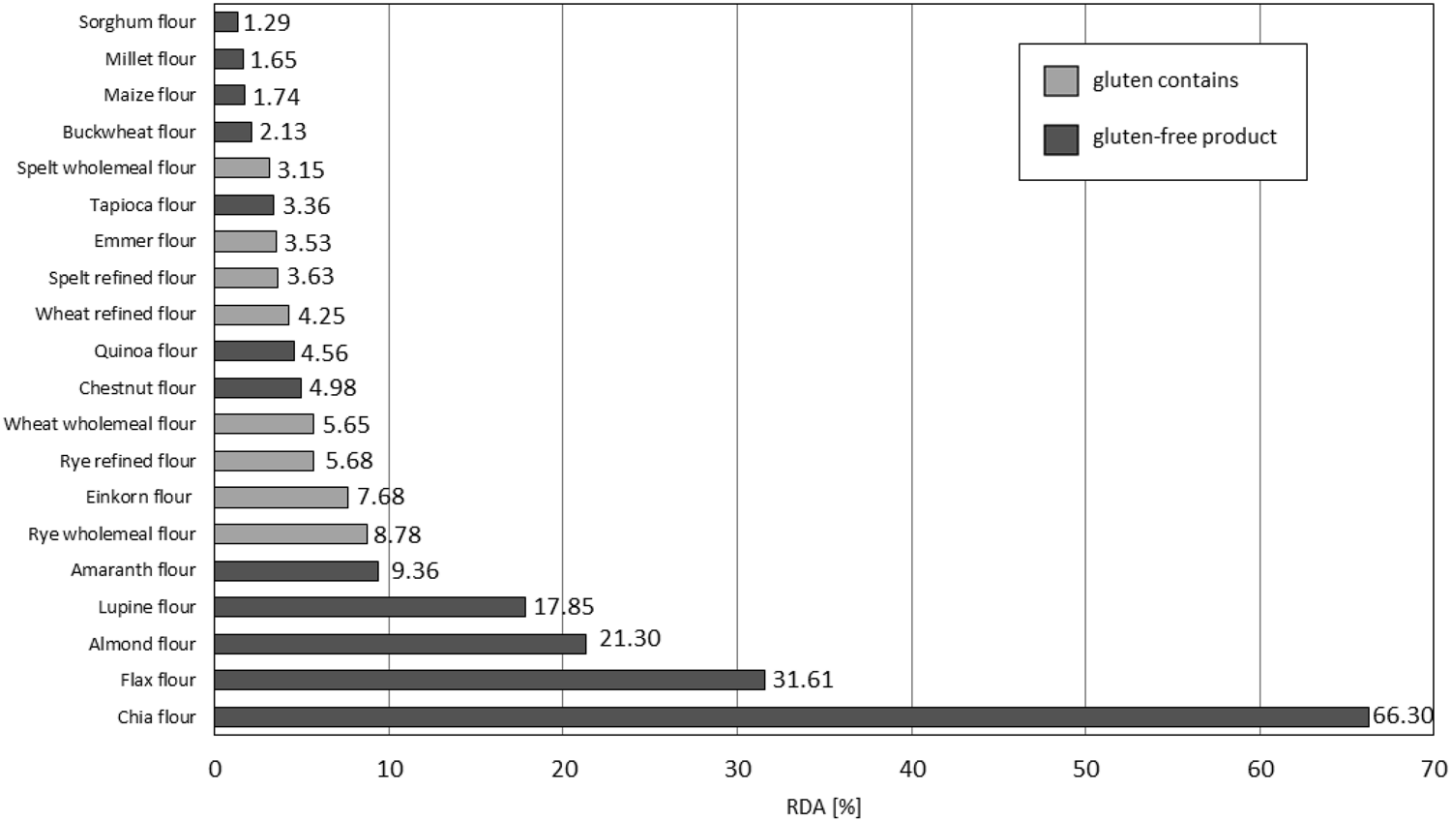
Assessment of the coverage of the daily requirement of calcium contained in 100 g of the analyzed flour for an adult.

On the basis of the daily reference intake values of minerals (Table 2), the percentage to which the consumption of 100 g of a given food product covers the average daily requirement for the element was calculated. The graph (Fig. 2) shows the data on the percentage realization of the reference intake values of calcium in gluten containing and gluten-free products.

**Table 2:** Daily reference intake values for minerals (for adults)^38^.

Macroelements				Microelements			
Ca	800 mg	Mg	375 mg	Fe	14 mg	Zn	10 mg

Wheat is the most popular cereal, and wheat flour is one of the dominant Polish exports in the grain sector^38^. It was found that 100 g of refined wheat flour, so called white which is the most popular type of flour, satisfies only 4.3% of the daily reference intake value for calcium (Fig. 2). Wholemeal wheat flour allows to supplement slightly more—5.7% of the RDA Ca. This is due to the fact that the minerals are concentrated in the peripheral layers of the grain. The processing of grains during the production of refined flour, such as the purification and milling process, results in lower content, and therefore calcium reference values, in refined flours than in wholemeal products. Consumption of 100 g of chia flour would cover as much as 66.3% of the RDA (Fig. 2). Various amounts of packaged chia seeds are available on the market for consumption, but the recommended daily intake is about 15–25 g DM/day^39^. In contrast, EFSA's NDA panel^40^ indicates that the maximum recommended intake of chia seeds is 15 g per day. Similarly, although the consumption of 100 g of a product prepared from flaxseed flour would cover more than 30% of the RDA (Fig. 2.), the amount of flaxseed consumed per day over an extended period of time can be 40 to 50 g^41,42^. Therefore, it is worth noting that the data shown in the graph (Fig. 2) were standardized for the same amount of the product analyzed, but it should be taken into account that a person wishing to overcome a calcium deficiency condition is not able to replace 100 g of wheat flour in a recipe with, for example, 100 g of chia or flax flour.

Magnesium was another macronutrient analyzed, with concentrations ranging from 14.6 to 590.75 mg/100 g (Table 1). Gluten-free flours (those derived from flax, chia, buckwheat, almond, amaranth and quinoa) were found to be highly rich in magnesium. Flax flour contains the most magnesium, i.e. 590.75 mg/100 g, followed by chia 499.2 mg/100 g (Table 1), and according to literature data there are lower Mg contents for flax from 350 to 431 mg/100 g^21,24^ and respectively for chia from 250 to 322 mg/100 g^19,21,22^. Buckwheat and almond flour are unusually rich in magnesium, as the concentrations of this element in the products were 233 and 229.8 mg/100 g, respectively (Table 1), a result similar to the 219 mg/100 g reported by Kunachowicz for buckwheat and 239 mg/100 g for almonds^26^. A slightly lower Mg content was determined by Markiewicz-Keszycka^43^. In contrast, Ikeda et al. reported that buckwheat flour contains as much as 264 ± 14 mg/100 g^29,30^. Amaranth flour is also characterized by a significant content of this macronutrient, amounting to 211.1 mg/100 g (Table 1). Numerous works have evaluated the mineral composition of amaranth flour or amaranth seeds, and the magnesium content has been in the range of 231–279.2 mg/100 g^21,26–28^. The results obtained for sorghum flour (54.1 ± 4.10 mg/100 g) and millet flour (85.5 ± 13.5 mg/100 g) are lower than the value suggested by the literature (116–164 mg/100 g)^19,26^. The magnesium content of chestnut flour was found to be slightly lower than the 68.8 and 74 mg/100 g reported by the USDA and Kunachowicz^19,26^. According to Kunachowicz^26^, the Mg content of the following flours: corn flour is 40 mg/100 g and tapioca flour is 2 mg/100 g, we show much higher values of 79.8 ± 0.15 and 14.6 ± 0.1 mg/100 g of these products, respectively. According to our determinations, flour derived from quinoa contains 147.8 mg of Mg per 100 g of product (Table 1), and interestingly, the content of this element in quinoa seeds is also higher according to other indications—in the range of 164- 230 mg/100 g dry weight^19,21,26–28^.

On the basis of the data (Table 1), it was estimated that the average content of Mg is characterized by gluten-free flours: whole wheat, einkorn and emmer. Among flours derived from cereals, a relatively high magnesium content can be indicated in whole wheat flour—105.5 mg/100 g, and a similar 100.3 mg/100 g – from einkorn. As we have shown in Table 1, it is small spelt flour that has the highest levels of magnesium relative to other wheat, similar to other researchers who have compared the content of this macronutrient in wheat grains^33,34^. The Mg content of wheat and wheat flour in the literature ranges from 35 to 140 mg/100 g^44,45^. Fernández-Canto indicated the content of this macronutrient at wheat wholemeal 123.1 ± 4.4 mg/100 g and wheat refined flour 39.6 ± 0.3 mg/100 g^31^. An even higher content was obtained by ECP-MS measurement—1.4 ± 0.2 g/kg (after conversion—140 mg/100 g) of the product^32^—perhaps putting them together in the range that other researchers have shown Mg in this product in the range: 123.1–140 mg/100 g^31,32^. It varies strongly with the type of flour of the same origin, however, formed after grinding cereal grains (0.21 ± 0.07 g/kg)^32^. However, the lowest concentrations of magnesium were found in refined wheat flour 25.2 mg/100 g and tapioca flour 14.6 mg/100 g (Table 1), which corresponds well with data according to the USDA, while Kunachowicz reports values for this element of 10 mg/100 g for wheat flour and 2 mg/100 g for tapioca, respectively^19,26^. The Mg content for refined rye flour of 38 ± 7.51 mg/100g is much lower than the values suggested by the USDA^19^ and Ertl et al.^32^. Similarly, for whole grain spelt flour, 70.1 mg/100 compared to 124–130 mg/100g^19,32^, while refined 35.0 ± 0.30 mg/100g is already close to the result suggested by Ertl et al. 40 mg/100g^32^.

The recommended daily allowance (RDA) for magnesium ranges from 250 to 420 mg per day according to various recommendations. The variability in RDA can be attributed to the presence of many dietary and physiological factors affecting the neutral determination of magnesium balance, such as weight, gender, age, calcium and phosphorus status, as well as protein and other antioxidant nutrients^44–47^. Long-term persistence of magnesium deficiency is common in treated CD patients, likely reflecting the low magnesium content of gluten-free grains products^48^.

**Figure 3:**
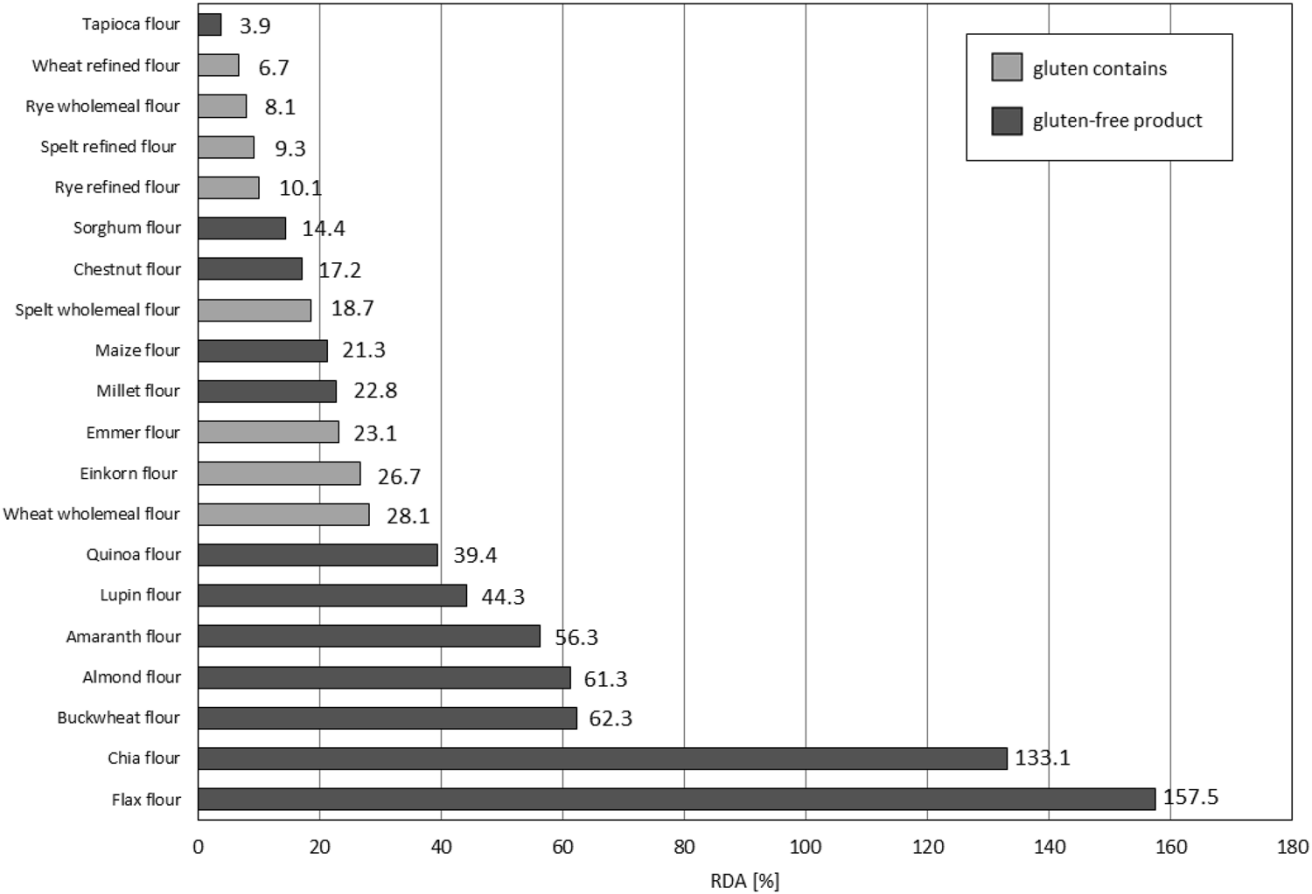
Assessment of the coverage of the daily requirement of magnesium contained in 100 g of the analyzed flour for an adult.

The magnesium content of flours derived from gluten-free products is much higher (Table 1) and meets an average of 52.8% of the daily requirement for this macronutrient in an adult. However, this value is again inflated by flax and chia flours (Fig. 3), for which the indicated values are only approximate, due to the reference to 100 g of product. Maintaining the recommendations for the maximum daily intake of the flours in question^39–42^, flax and chia flours would cover 28.57% and 11.27% of the average reference values for magnesium, respectively (Fig. 3). It can be seen that GFPs such as flours from: buckwheat, almond, amaranth, lupin and quinoa secure about 50% of the daily portion required for magnesium, while among gluten-free flours, whole milled from classic wheat and ancient varieties – emmer and einkorn—lead the way (Fig. 3). However, even after converting the Mg requirement into smaller doses, gluten-free flours cover almost twice the percentage of its RDA than flours derived from gluten-containig seeds (Fig. 3).

In our study, the iron content of the flours analyzed ranged from 0.59 to 13.295 mg/100 g of product (Table 1). The highest Fe content is represented by flours: flax 13.29 mg/100 g and chia 6.63 mg/100 g (Table 1), which also corresponds to the USDA data, except that a value of 7.72 mg/100 g is given for chia. The literature reports lower iron concentrations in flax flour, ranging from 4.11 to 5.78 mg/100 g^19,21,24^. In contrast, chia has a wide range of Fe content from 5.3 to 24.3 mg/100 g in other studies^19,21,49^. Next in our Fe determinations decreasingly, but still at similar levels, are wholemeal rye, wholemeal wheat, quinoa, amaranth, einkorn, and refined rye flours (Table 1). Researchers report higher Fe content data for amaranth flour 6.74- 9.62 and quinoa flour 4.87- 8.9 mg/100 g^21,26,28^. The USDA and Kunachowicz report that buckwheat flour is rich in iron, with the content occurring at 3.84–4 mg/100 g of product, which is higher than the value obtained in our analysis (2.54 mg/100 g) (Table 1)^19,26^. In contrast, the lowest concentrations of iron were found in flours made from tapioca, chestnut, sorghum, common spelt and corn (Table 1). Extremely poor in iron is tapioca flour 0.59 mg/100 g (Table1), which actually correlates with USDA data of 0.27 mg/100 g, while Kunachowicz reports a value of 1.3 mg/100 g twice as high^19,26^ and Bayata 0.27 mg/100 g^50^. Chestnut flour, which amounted to 1.18 ± 0.05 mg/100 g, is slightly lower than the USDA suggested value of 1.62 mg/100 g^19^. According to Table 1, the amounts of this element in sorghum, almond and quinoa flours appear to be similar to literature values^19,21^. Only Kunachowicz^26^ reports for GF flours: from amaranth, buckwheat, sorghum, millet, chestnut, corn and tapioca (7.2; 4.0; 4.4; 3.7; 3.0; 2.4 and 1.3 mg/100 g of these products, respectively), which exceeds the values from our determinations (Table 1).

Among gluten-containing flours, the highest concentrations were found in wholemeal rye flour and wholemeal wheat flour—5.03 mg/100 g and 4.77 mg/100, respectively (Table 1). As in the case of macronutrients, it can be indicated that among seeds flours, wholemeal products are richer in iron content than their purified forms. Comparing the results of iron content in different types of gluten-containing flours with literature data, some differences can be observed. Wheat flour—refined and whole grain, respectively—is characterized by a higher content of the micronutrient (2.10 ± 0.95 mg/100 g and 4.77 ± 0.35 mg/100 g) than in the literature (7.1 ± 3.2 mg/kg and 34 ± 3 mg/kg )^32^. In the case of spelt flour, the Fe for refined flour of 1.87 ± 0.05 mg/100 g appears to be slightly higher than the values suggested by the literature of 14 ± 2 mg/kg^32^, while that for whole grain flour of 2.32 ± 1.24 mg/100 g is lower than the 39 ± 2 mg/kg for wholemeal)^32^. Of the various wheat, Biel et al. found^51^ that it was spelt that stood out for having the highest Fe levels, as in Zhao et al.^52^, it was spelt and emmer that stood out for having the highest levels of this element, even compared to another ancient wheat, einkorn. Hidalgo and Brandolini^33^ indicated that the Fe content of einkorn flour ranged from 37.2 to 62.6 mg/kg, and our result falls within this range. For rye flour, the results obtained by ICP-OES analysis for refined flour (4.01 ± 1.52 mg/100 g) are higher than the value suggested by the literature (about 2.54 mg/100 g^19^), while the results for wholemeal rye flour (5.03 ± 1.15 mg/100 g) are close to the literature values (20 ± 1 mg/kg)^32^.

Iron deficiency anemia affects about one-third of the world's population, and about half of the cases are due to dietary iron deficiency, which is associated with adherence to vegan and vegetarian diets, among other factors, moreover, this low iron status may be reduced by the observed low consumption of legumes and cereals^53–55^.

**Figure 4:**
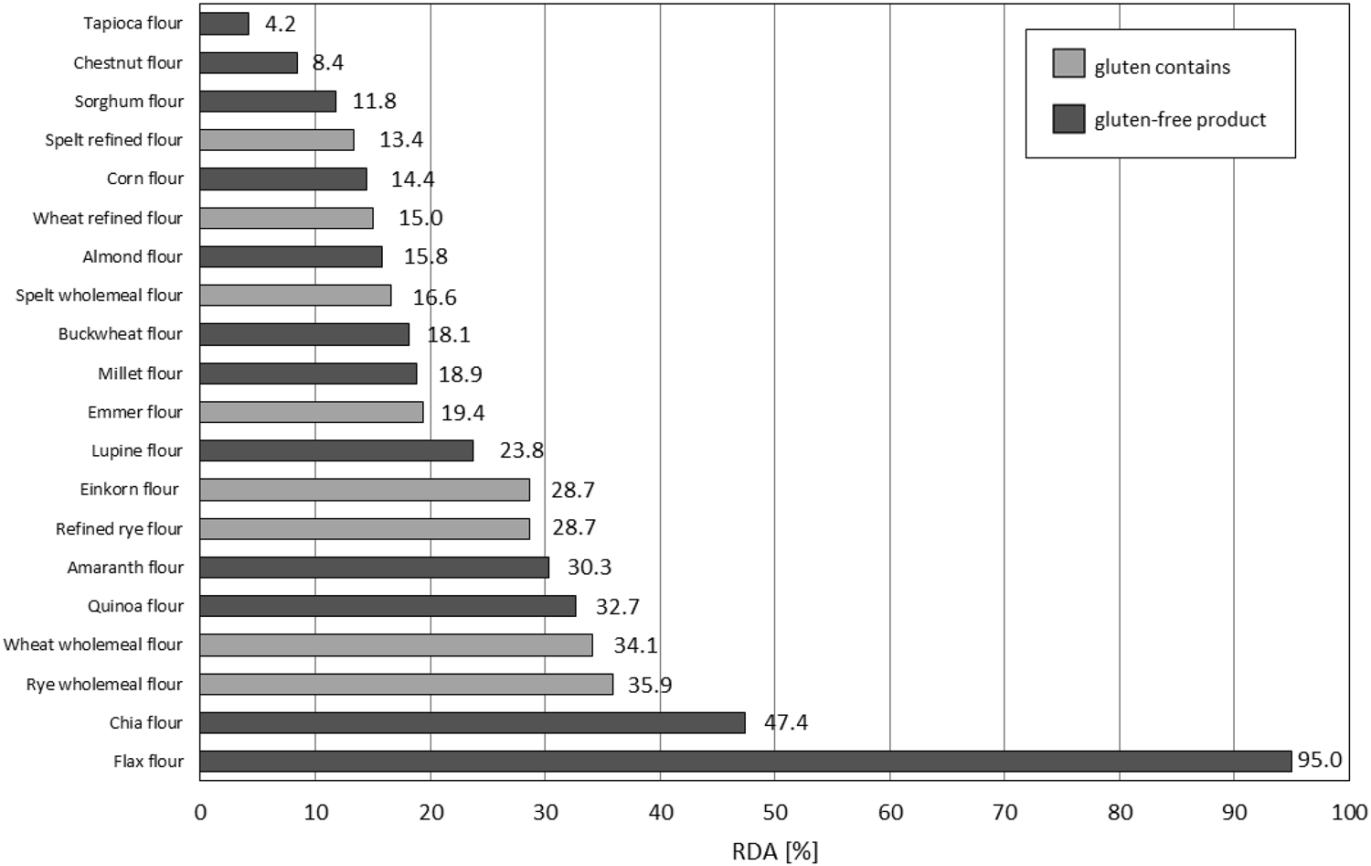
Assessment of the coverage of the daily requirement of iron contained in 100 g of the analyzed flour for an adult.

The average iron coverage for all flours analyzed was 25.62%. Again, the highest values can be found for flax and chia flours (Fig. 4). This time, however, it is important to note the high coverage of this micronutrient in wholemeal flours—rye and wheat (Fig. 4). Therefore, it is important that the GFD diet abounds with products that will replace the deficiencies of this element, after the withdrawal of classic grains. Gluten-free flours—derived from quinoa or amaranth—may prove to be a solution—they have only slightly lower %RDA values (Fig. 4).

Zinc contents in the flours tested ranged from 0.125 to 6.44 mg/100 g (Table 1). We also showed the highest zinc content for flax and chia- 6.44 and 6.12 mg/100 g, respectively (Table 1), which is in agreement with the determinations of other researchers, who determined zinc in these plants in the range of 4.58–7.74 mg/100 g^19,21,49^. In flax seeds, Zn was recorded at a level of 2.7–3.3 mg/100 g^23,24^. Zinc was determined at an average level in ground products from: almonds, emmer, rye, wheat and lupin, followed by: millet, buckwheat, quinoa and amaranth (Table 1). It is evident that whole-grain products contain more of this element than those in which grains have been refined and processed during fine milling. The levels of Zn in wheat flour and refined and whole-grain rye flour were 0.76 and 1.47 and 2.73 and 3.25 mg/100 g, respectively (Table 1). These values are similar to those reported by Ertl and Goessler^32^, in whom, however, whole wheat flour was characterized by a slightly higher content of this micronutrient (39 ± 6 mg/kg), and wholemealrye flour by 30 ± 2 mg/kg. For refined and whole-grain spelt flour, the zinc content (1.33 ± 0.05 mg/100 g) is slightly lower than the values reported in the literature—17 ± 1 mg/kg for refined flour^32^ and 3.59 mg/100 g for whole-grain^19^. Genc and MacDonald^56^ also reported higher values for this element in ancient grains—especially emmer, similar to ours, where we find the most zinc among wheat in einkorn, followed by emmer, relative to refined wheat flour [Table 1]. Similar values for this micronutrient in einkorn grains are indicated by Hidalgo and Brandolini^33^. Buckwheat, chestnut and quinoa flours correspond to the contents reported by the USDA database^19^. However, the elemental content results for sorghum and amaranth flour are slightly lower than the literature value (1.66 mg/100 g and 3 mg/100 g, respectively) and higher for almond flour (2.8 mg/100 g)^19^. Flour from millet, buckwheat and lupin show similar contents of this element (Table 1), but other researchers reported wider ranges for Zn 0.73–4.2 mg/100 g, depending on the millet variety, which they also linked to growing conditions^57^. As for lupin flour, the Zn content is at 2.46, which correlates well with the results of others^58,59^. Higher values for zinc are also found in statements according to Kunachowicz^60^, where buckwheat and millet flours have zinc contents of 3.75 and 3.66 mg/100 g, respectively. According to a compilation by Multari et al.^61^ buckwheat and wheat flours have this micronutrient at the level we determined, while in lupin flour the researchers show Zn at a slightly higher level of 3.65 mg/100 g of product. Tapioca, refined wheat flour, sorghum, chestnut and refined spelt flour were characterized by the lowest Zn concentrations (Table 1), which also correlates properly with the tables according to Kunachowicz and others^50,60^.

Patients with gastrointestinal disorders, including celiac disease, have a problem with zinc deficiency. Decreased plasma zinc levels have been observed in both untreated CD patients and patients in clinical remission. Zinc deficiency is known to correlate strongly with intestinal villous atrophy. While serum zinc levels are significantly lower in children with untreated CD and enteropathy, they normalize after transition to GFD. There are wide discrepancies between dietary recommendations for zinc set by different expert groups. Improving the level of zinc in the food consumed may have an impact on reducing the incidence of some gastrointestinal diseases, so research on the content of this mineral in food seems warranted^62,63^

**Figure 5:**
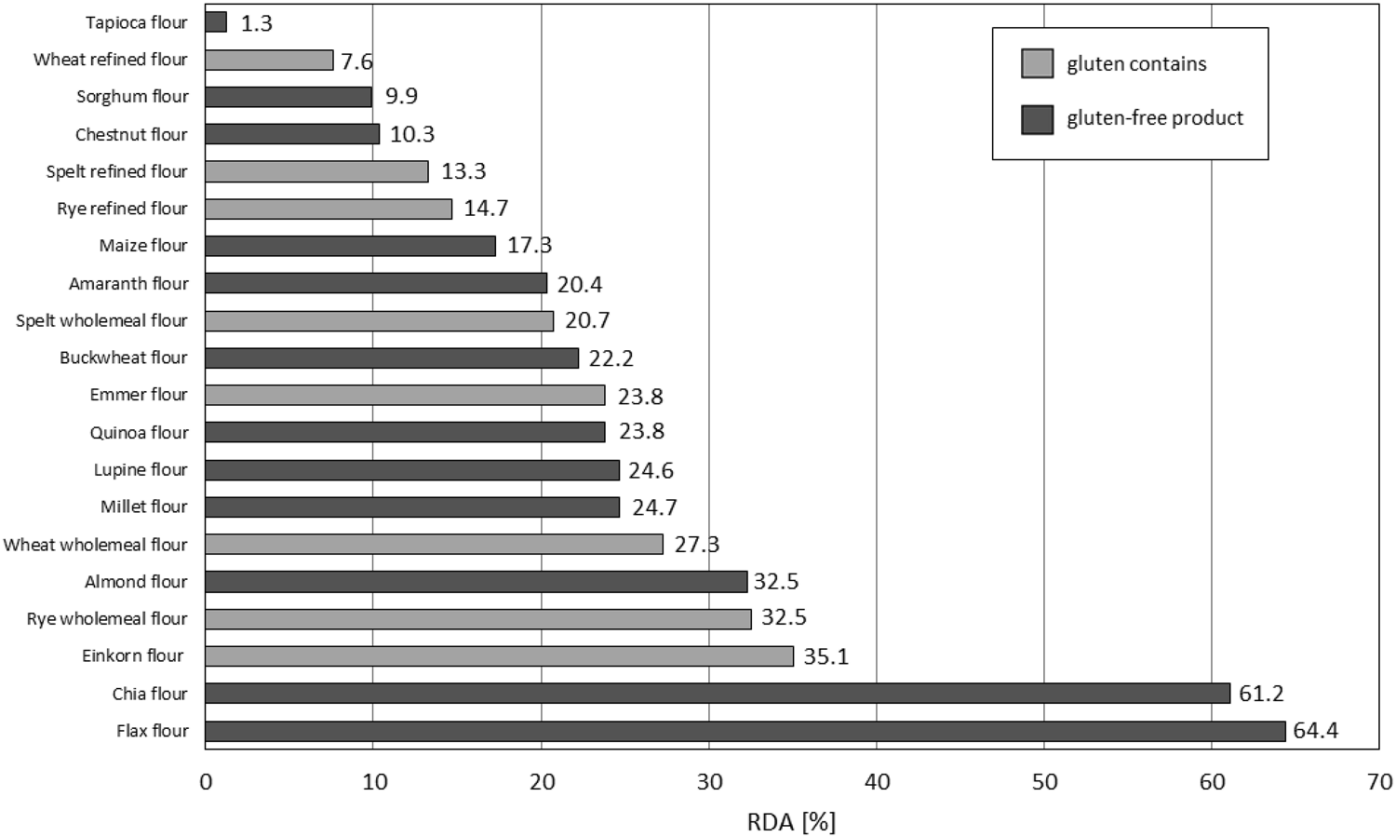
Assessment of the coverage of the daily requirement of zinc contained in 100 g of the analyzed flour for an adult.

A summary of the data on zinc levels in gluten and gluten-free products shows that wholemeal rye and ancient einkorn flours contribute the most to the daily requirement for this mineral, and is similar to the calculated %RDA for almond flour, which is about half that of chia and flax flours (Fig. 5). Among gluten-free flours, amaranth, buckwheat, quinoa, lupin and millet flours cover from 20 to 25% of the daily requirement for this element in the analyzed dose of 100g of product (Fig. 5).Similar ranges characterize wholemeal wheat flour and its ancient variety emmer wheat (Fig. 5). Refined wheat flour meets the demand for this element at 3.6 times less than its whole-grain counterpart (Fig. 5), which shows the importance of consuming whole-grain products.

**Figure 6:**
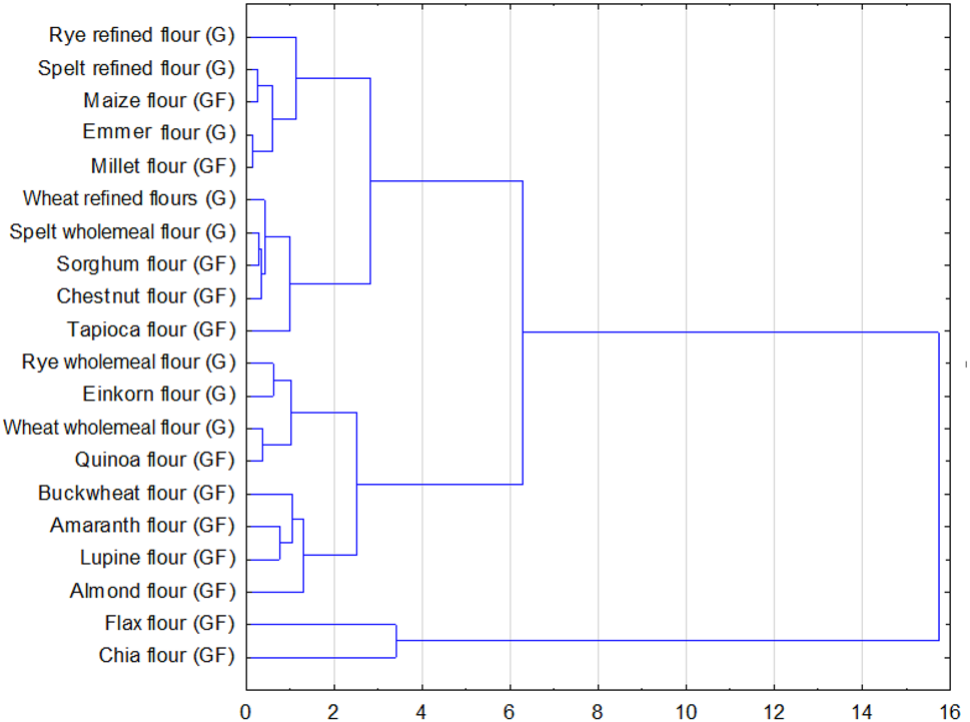
Dendrogram of the tested flours based on mineral content: Ca, Mg, Fe, Zn, (G)—contains gluten, (GF)—gluten-free product.

For a more comprehensive study of the amounts of mineral components determined for all gluten-containing and gluten-free flours, the data were subjected to statistical analysis—hierarchical cluster analysis (HCA), using Ward's Method agglomeration and Euclidean distance. According to the cluster analysis of G and GF flours based on the content of 4 minerals, the flour samples were divided into three groups called clusters. The WARD method was used as a clustering algorithm, which determines for each cluster the average of each variable, and also the distance between clusters is determined as the average of the distance from the center element to all elements of the other clusters of another cluster. The analysis made it possible to distinguish three groups of flours differing in the level of analyzed mineral components (Fig. 6). The first cluster included 10 flours under study: millet flour, chestnut flour, corn flour, refined spelt flour, whole grain spelt flour, emmer wheat flour, sorghum flour, tapioca flour, refined rye flour and refined wheat flours. In the second cluster, 8 flours were identified: amaranth flour, buckwheat flour, lupine flour, almond flour, quinoa flour, einkorn flour, wholemeal rye flour and wholemeal wheat flour. The third group consisted of only two flours: chia flour and flax flour (Fig. 6), as was evident from the detailed graphs for each mineral (Figs.2, 3, 4, 5).

**Figure 7:**
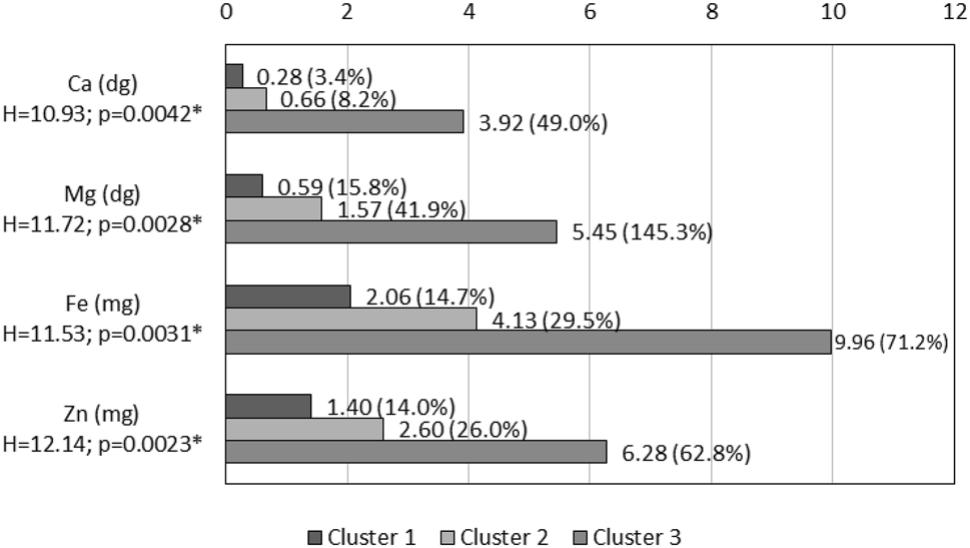
Median of selected flour minerals by concentration (1dg = 0.1g; 1mg = 0.001 g, H-value of Kruskall-Wallis test; *-significant variation at *p* < 0.05, RDA is written in parentheses).

Figure 7 shows what the average amount of minerals for each cluster looks like, which compares well with the results of individual minerals obtained for different flours (Figs. 2, 3, 4, 5). The level of all four minerals tested (Ca, Mg, Fe, Zn) was significantly highest in flours belonging to cluster three (392 mg, 545 mg, 9.96 mg, 6.28 mg—respectively) and significantly lowest in flours of cluster one (28 mg, 59 mg, 2.06 mg, 1.40 mg—respectively). In the third agglomeration, represented by flours: flax and chia, the RDA level in all analyzed elements was almost 50% (from 49.0%—for Ca to 145.3%—for Mg). The RDA level in agglomeration two was from 8.2%—in the case of Ca to 41.9%—in the case of Mg. The level of RDA in agglomeration one accounted for up to 3.4%—for Ca to 15.8%—for Mg (Fig. 7).

## Conclusions

Our results have shown that most gluten-free flours such as: buckwheat, almond, amaranth or quinoa are high in Ca, Mg, Fe and Zn contents and can be a healthy alternative in the GFD. Amaranth, lupin and almond flour are particularly valuable for calcium deficiency, while the three flours mentioned above and buckwheat are particularly valuable for magnesium deficiency. Among other things, almond flour has a high zinc content, while amaranth and quinoa flours are relatively rich in iron, which is comparable to wholemeal flours made from wheat and rye. Chia and flax flours scored particularly well in our analysis, and flax and chia also have higher levels of bioelements according to the US Department of Agriculture compilations, in particular: P, Fe, Ca and Zn, than conventional oilseeds and grains^19^. Despite the health benefits, making bread from gluten-free flours is technologically challenging, as the lack of gluten affects the structure and elasticity of the dough. To achieve similar properties, blends of different gluten-free flours and other additives are used^13,50,58,64^. Our work complements analytical data on macronutrients and micronutrients in gluten-free products, which may be an increasingly popular part of the GFD for patients with celiac disease, allergy and gluten sensitivity. Our findings provide valuable information on the nutritional value of gluten-free flours, supporting both consumers and manufacturers in creating healthy and tasty gluten-free products.

## Data Availability

All data generated or analysed during this study are included in this published article.
